# Combining the Photocatalysis and Absorption Properties of Core-Shell Cu-BTC@TiO_2_ Microspheres: Highly Efficient Desulfurization of Thiophenic Compounds from Fuel

**DOI:** 10.3390/ma11112209

**Published:** 2018-11-07

**Authors:** Jing Liu, Xiao-Min Li, Jing He, Lu-Ying Wang, Jian-Du Lei

**Affiliations:** 1 Beijing Key Laboratory of Lignocellulosic Chemistry, College of Materials Science and Technology, Beijing Forestry University, Beijing 100083, China; liujing@bjfu.edu.cn (J.L.); lixiaomin0128@126.com (X.-M.L.); hejing2008@sina.com (J.H.); wangly@bjfu.edu.cn (L.-Y.W.); 2 MOE Key Laboratory of Wooden Material Science and Application, Beijing Forestry University, Beijing 100083, China

**Keywords:** photocatalysis, absorption, TiO_2_, Cu-BTC, desulfurization

## Abstract

A core-shell Cu-benzene-1,3,5-tricarboxylic acid (Cu-BTC)@TiO_2_ was successfully synthesized for photocatalysis-assisted adsorptive desulfurization to improve adsorptive desulfurization (ADS) performance. Under ultraviolet (UV) light irradiation, the TiO_2_ shell on the surface of Cu-BTC achieved photocatalytic oxidation of thiophenic S-compounds, and the Cu-BTC core adsorbed the oxidation products (sulfoxides and sulfones). The photocatalyst and adsorbent were combined using a distinct core-shell structure. The morphology and structure of the fabricated Cu-BTC@TiO_2_ microspheres were verified by scanning electron microscopy, high-resolution transmission electron microscopy, energy-dispersive x-ray spectroscopy, X-ray powder diffraction, nitrogen adsorption-desorption and X-ray photoelectron spectroscopy analyses. A potential formation mechanism of Cu-BTC@TiO_2_ is proposed based on complementary experiments. The sulfur removal efficiency of the microspheres was evaluated by selective adsorption of benzothiophene (BT) and dibenzothiophene (DBT) from a model fuel with a sulfur concentration of 1000 ppmw. Within a reaction time of 20 min, the BT and DBT conversion reached 86% and 95%, respectively, and achieved ADS capacities of 63.76 and 59.39 mg/g, respectively. The BT conversion and DBT conversion obtained using Cu-BTC@TiO_2_ was 6.5 and 4.6 times higher, respectively, than that obtained using Cu-BTC. A desulfurization mechanism was proposed, the interaction between thiophenic sulfur compounds and Cu-BTC@TiO_2_ microspheres was discussed, and the kinetic behavior was analyzed.

## 1. Introduction

Sulfur compounds in fuel oils present a major air pollution problem because of the sulfur oxide content (SOx, x = 2, 3) in the exhausted gasses. These oxides contribute to acid rain and acid smog, and they are also harmful to human health [1]. Removal of sulfur compounds from petroleum fractions is an urgent concern, as it relates to producing clean fuels and reducing environmental pollution. The commercial desulfurization technology that is currently used in refineries is hydrodesulfurization (HDS), which is operated at high temperatures (about 350 °C) and H_2_ pressures (2–10 MPa) [2]. HDS is an efficient method for thiophene (Th), benzothiophene (BT), and dibenzothiophene (DBT), but not for DBT alkyl derivatives such as 4,6-dimethyldibenzothiophene (4,6-DMDBT) under normal operating conditions [3,4,5]. Oxidation of these compounds can occur under even the most mildly reactive conditions [6], but the organosulfur compounds, oxidation products and solvent seems to be separated not easily.

Adsorptive desulfurization (ADS) provides several advantages, such as deep removal of organosulfur compounds under ambient conditions. As such, ADS can be applied in low-sulfur fuel production, which mainly involves adsorbents [7]. Various adsorbents have been developed for the ADS of transportation fuels. These materials include active carbons [8], zeolites [9,10], alumina [11], titanium oxide [12], zinc oxide [13], and mixed metal oxides [14], among others [15,16]. Metal organic frameworks (MOFs) with porous hybrid inorganic-organic frameworks have recently been employed in deep desulfurization [17]. For instance, the porous framework of HKUST-1 (also referred to as Cu-BTC, Hong Kong University of Science and Technology), with Cu_3_(BTC)_2_ as a formula unit is a widely known MOF that has high surface area and pore volume; it exhibits superior characteristics suitable for certain processes, such as gas storage, separation, catalysis, and magnetization [18]. Hong-Xing Zhang et al. [19] compared the adsorption behavior of Cu-BTC, Cr-BTC, Cr-BDC, and Cu-BDC for thiophenic sulfur in model diesel oil; Cu-BTC exhibited the highest adsorption capacity for DBT. The adsorption mechanism underlying of MOFs is influenced by a number of factors, including the suitability of the components of the framework, the pore size and shape, and the exposed Lewis acid sites that match the S-compound to be adsorbed. Most MOFs consist of highly-ordered 3D networks, large pore volumes, and high specific surface areas [20]; however, the polyaromatic hydrocarbons that are highly concentrated in real diesel fuel exhibit low ADS selectivity. Sulfur compound-adsorbent interaction is considered a crucial parameter for high desulfurization selectivity [21].

Studies have indicated that the conversion of thiophenic compounds to sulfones in an adsorbent material may effectively improve ADS selectivity [22]. In recent years, the photocatalytic oxidation of sulfur compounds that use neat and structural modified TiO_2_ under ultraviolet (UV) irradiation has drawn significant interest [23,24,25]. Xueni Sun et al. [26] evaluated the removal of thiophenic S-compounds from hydrocarbon fuels under UV light irradiation over TiO_2_ and Ag/TiO_2_ adsorbents. Consequently, the Ag/TiO_2_ adsorbent obtained the highest removal capacity at 6.35 mg S/g adsorbent in UV-aided ADS using a model fuel with 1000 ppmw H_2_O. The sulfur removal performance of TiO_2_ was also assessed after a one-time ex-situ UV-treatment that used BT in n-octane as the model fuel. Ex-situ photo-treatment prior to adsorption was found to improve the desulfurization performance of TiO_2_. TiO_2_ exhibited a high sulfur removal capacity at 5.95 mg S/g adsorbent following a single ex-situ UV-treatment before ADS [27].

The Cu-MOFs and TiO_2_ composites has been used in oxidation, decolorization, photocatalysis. For example, Lijuan Shen et al. [28] synthesized a porous Cu_x_O/TiO_2_ material by a MOF-templated strategy which showed excellent catalytic activity for CO oxidation. They found that metal oxide composite could enhance dispersion of the active phase. Binary photocatalytic composites (TiO_2_/Cu-BTC) were tested for the decolorization of methylene blue (MB) and methyl orange (MO), and Cu-BTC based composites show higher dark adsorption abilities and higher degradation efficiencies toward MB [29]. V. Ramasubbu et al. [30] synthesized a TiO_2_ aerogel-Cu-BTC composite which showed a significant increase of absorption in the visible region. The incorporation of Cu-BTC to TiO_2_ aerogel are promising materials for photovoltaic and photocatalytic applications. A new type of photocatalyst for selective aerobic oxidation of benzylic alcohols was developed, which was prepared through incorporation of amorphous TiO_2_ within the prepared mesoporous Cu-BTC. This photocatalyst showed high selectivities (93–99%) with moderate to high conversions (32–100%) [31]. Furthermore, TiO_2_ and Cu-BTC were successfully coupled via sol-gel method to form a hybrid porous nanocomposite (PNC) which showed highly photoactive under UV irradiation. Combined with the photocatalytic activities and water vapor adsorption capacities such hybrid porous structures can be developed and applied to environmental systems [32]. Hongmei Wang et al. [33] synthesized a core-shell structured photocatalyst with functional Cu-BTC as core and porous ultrathin anatase film as shell, and evaluate the photocatalytic performances by isopropanol degradation experiments. The experimental results revealed that Cu-BTC can provide a special pathway for photogenerated electrons migration and thus restrain the recombination of electrons and holes to increase the photocatalytic efficiency. However, core-shell Cu-BTC@TiO_2_ has not been applied in photocatalysis-assisted adsorptive desulfurization.

To improve desulfurization, desulfurization typically combines the photocatalyst and the adsorbent that selectively remove organosulfur compounds by integrating selective oxidation and solid adsorption. Photocatalysis and adsorption were used together to achieve rapid and deep desulfurization for clean fuel production [34]. In the current study, Cu-BTC@TiO_2_ microspheres were prepared, with the photocatalyst TiO_2_ as the shell and Cu-BTC as the core. The TiO_2_ shell on the surface of the Cu-BTC achieved the photocatalytic oxidation of thiophenic S-compounds (BT and DBT), and the Cu-BTC core adsorbed the oxidation products (i.e., the corresponding sulfoxides and sulfones). These microspheres simultaneously separated the catalysts and products via deep desulfurization.

## 2. Materials and Methods 

### 2.1. Materials

Benzothiophene (BT, 98%) and dibenzothiophene (DBT, 98%) were purchased from Shanghai D&B Chemical Technology Co., Ltd., China. Copper (II) nitrate trihydrate [Cu(NO_3_)_2_·3H_2_O, 99%] and tetrabutylortotitanate (TBOT, 99%) were supplied by Sinopharm Chemical Reagent Co., Ltd., Beijing, China. Trimesic acid [C_6_H_3_(COOH)_3_, 99%] and polyvinyl pyrrolidone (PVP, 99%, Mr 40000) were provided by J&K Scientific Ltd., Shanghai, China. All other reagents were commercially available and used without further purification.

### 2.2. Synthesis

The Cu-BTC@TiO_2_ microspheres were prepared in two steps: (1) The Cu-BTC core was synthesized as described in the literature [35]. Cu(NO_3_)_2_·3H_2_O (0.9 g) and polyvinyl pyrrolidone (0.4 g) were initially dissolved in 50 mL methanol and then stirred to obtain a transparent blue solution. With the use of a dropper, a 50 mL methanol solution with 0.43 g C_6_H_3_(COOH)_3_ was added dropwise into the aforementioned transparent solution to form a blue colloidal suspension. The colloidal solution was then continuously stirred at 400 rpm for 10 min and aged without interruption for another period of 24 h at 25 °C. The obtained blue precipitate was subjected to centrifugation, washed with methanol three times, and oven-dried at 60 °C. (2) A TiO_2_ shell was prepared using a versatile kinetics-controlled coating technique [36]. An ethanol dispersion of Cu-BTC core particles (100 mL, 0.3 mg/mL) was added into a conical flask charged with a concentrated ammonia solution (28 wt %, 0.3 mL) under ultrasound for 15 min. About 0.75 mL of TBOT was added dropwise within 5 min. The reaction continued for 24 h at 45 °C with continuous stirring. The resultant product was separated and then washed with deionized water and ethanol 3 times. Cu-BTC@TiO_2_ microspheres were obtained. The powders were dried at 60 °C overnight and calcined in air at 300 °C for 3 h before use.

### 2.3. Characterization

The morphology of the Cu-BTC@TiO_2_ microspheres was assessed by field-emission scanning electron microscopy (FESEM, SU8010, Hitachi, Tokyo, Japan), transmission electron microscopy (TEM, JEM-1010, JEOL, Tokyo, Japan), and high-resolution transmission electron microscopy (HRTEM, JEM-2100F, JEOL, Tokyo, Japan). For FESEM, acceleration voltage and working voltage were both 5 kV. TEM and HRTEM were operated with an accelerating voltage of 200 kV (point resolution of 0.23 nm). All the tested samples were sonicated in ethanol about 10 min, and then a drop of this dispersed suspension was placed on a carbon film-coated Cu grid (pore size 74 μm). The as formed sample grid was dried naturally under ambient conditions before insertion into the sample holder. Energy-dispersive x-ray spectroscopy (EDS) mapping was performed on an FEI Quanta FEG 250 and JEM-2100F instrument (JEOL, Tokyo, Japan). The crystallographic structures of the Cu-BTC@TiO_2_ microspheres were verified by X-ray powder diffraction (XRD, Bruker, karlsruhe, Germany). XRD patterns were recorded between the range of 5° and 50° 2θ by step scanning on a Bruker D8 Advance X-ray powder diffractometer equipped with a Cu-Kα source (40 kV, 40 mA) at a scanning rate of 5°/min. The surface areas of the microspheres were obtained by analysis of a nitrogen adsorption-desorption isotherm via the Brunauer Emmett Teller (BET) method on a V-Sorb 2800P surface area and pore distribution analyzer (Gold APP Instruments Corporation, Beijing, China). The microspheres were degassed at 180 °C for 10 h and analyzed with N_2_ at 77 K. X-ray photoelectron spectroscopy (XPS) data were collected using an ESCALab220i-XL electron spectrometer (VG Scientific, Thermo Fisher Scientific, Waltham, MA, USA) and 300W AlK_α_ radiation. The base pressure was approximately 3 × 10^−9^ mbar. The binding energy references to the C1s line were set to 284.8 eV from adventitious carbon.

### 2.4. Measurement of Desulfurization Performance

The desulfurization performance of the Cu-BTC@TiO_2_ microspheres was evaluated using a 100 mL photochemical reactor (Shanghai jiguang special lighting electric factory, Shanghai, China, see Figure 9) with air bubbled in a constant flow (about 60 L/h) as an oxidant. An appropriate amount of thiophenic S-compound (BT or DBT) was first dissolved into n-octane to be used as a model fuel wifth a sulfur concentration of 1000 ppmw. The light source used was a 300 W high-pressure mercury lamp (Shanghai jiguang special lighting electric factory, Shanghai, China) in a cylindrical quartz reactor equipped with a circulating water jacket. The first step in a typical operation involved heating the water bath to the desired reaction temperature (80 °C), followed by stabilization and adding 10 mL of the model fuel to the reactor. Microspheres were introduced into the reactor, followed by stirring. Samples were taken from the reactor after 5, 10, 15, 20, 30, 60, and 90 min to monitor the progress of the reaction. The microspheres were separated from the reaction system by centrifugation at 5000 rpm for 10 min. The products and byproducts were then analyzed by gas chromatography (GC, Agilent 7890B, Agilent Technologies, Santa Clara, CA, USA) equipped with a capillary column (HP-5 column, 30 m × 0.32 mm; carrier gas N_2_) and a flame ionization detector (FID, Agilent Technologies, Santa Clara, CA, USA).The injection temperature is 240 °C, injection volume is 2 μL, and split ratio is 50: 1. The oven temperature was initially set to 50 °C for 1 min and then heated at 25 °C/min to 280 °C for 5 min at 280 °C. Gas chromatography-mass spectrometry (GC-MS, Agilent Technologies, Santa Clara, CA, USA) with a gas chromatograph (Agilent GC-7890A, HP-5 column, Agilent Technologies, Santa Clara, CA, USA) and a mass spectrometer (Agilent MSD 5975C, Agilent Technologies, Santa Clara, CA, USA) was employed to determine the reaction products. A calibration with standard solutions (BT or DBT in octane) from 0 to 2000 ppm was used for quantification of the BT and DBT conversions. The standard curve equation of BT and DBT was Y = 1.3036 X + 1.6912 (R^2^ = 0.9993) and Y = 2.0798 X − 27.369 (R^2^ = 0.9996), respectively, where Y is the peak area and X is the concentration of BT/DBT (ppm). BT and DBT conversions were calculated as follows:

C = 100% − C_t_/C_0_ (1)

where C_0_ denotes the BT or DBT concentration at the start of irradiation, and C_t_ is the BT or DBT concentration at the sampling time.

## 3. Results and Discussion

### 3.1. Characterization of the Cu-BTC@TiO_2_ Microsphere

**Figure 1:**
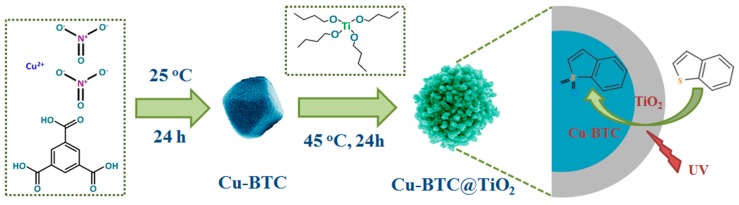
Schematic of mild synthesis of Cu-BTC@TiO_2_ microspheres and their desulfurization performance.

The synthetic method for the preparation of the Cu-BTC@TiO_2_ microspheres is presented in Figure 1. Cu-BTC was first prepared and then a TiO_2_ layer was deposited on the Cu-BTC surface to form the Cu-BTC@TiO_2_ microsphere via the TBOT sol-gel method. Under UV light irradiation with Cu-BTC@TiO_2_ photocatalysts, the oxidation of the thiophenic compounds was catalyzed into corresponding sulfones. Owing to the strong adsorption of the transformed sulfones into the inside of the Cu-BTC@TiO_2_ microspheres, thiophenic compounds were selectively removed from the model fuel.

**Figure 2:**
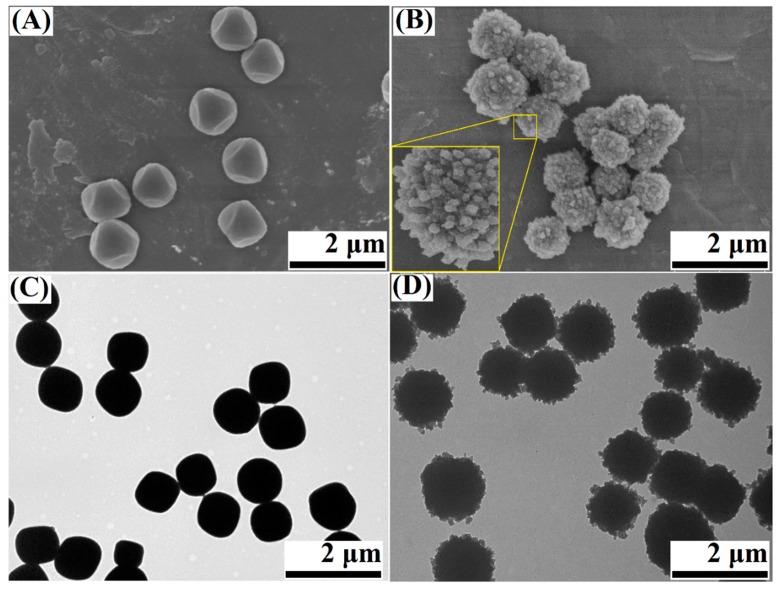
Field-emission scanning electron microscopy (FESEM) images (**A**,**B**) and TEM images (**C**,**D**) of Cu-BTC (**A**,**C**) and Cu-BTC@TiO_2_ (**B**,**D**); a magnified microsphere surface is shown as an inset in (**B**).

**Figure 3:**
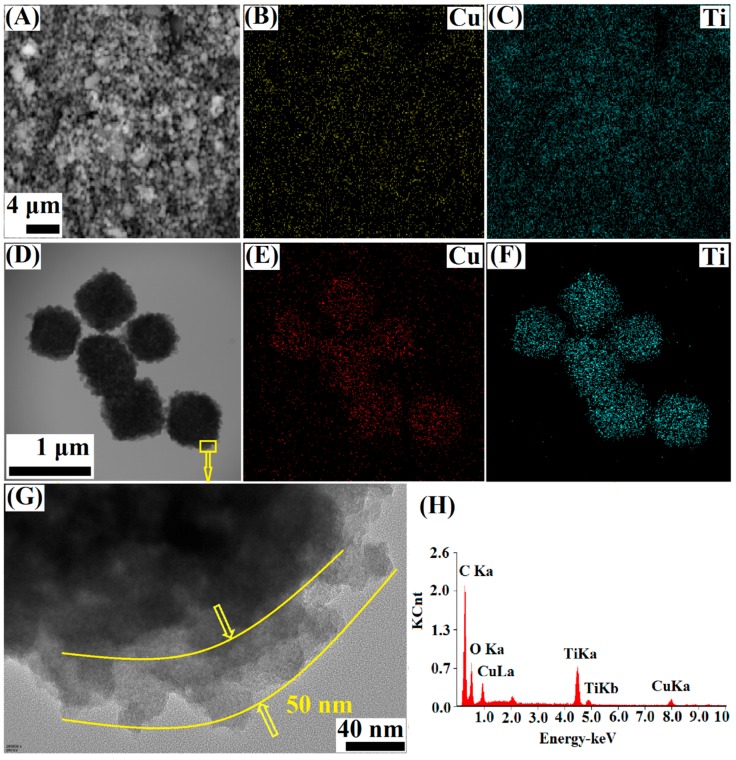
SEM (**A**–**C**), TEM (**D**–**F**), and high-resolution transmission electron microscopy (HRTEM) (**G**) images and corresponding energy dispersive spectroscopy mapping images of Cu-BTC@TiO_2_.

Figure 2 presents the typical FESEM and TEM images of the Cu-BTC and Cu-BTC@TiO_2_ microspheres. Uniform Cu-BTC microparticles with a diameter of approximately 0.5 μm as the core were synthesized (Figure 2A,C). Then, a TiO_2_ particulate layer was coated on the surface of the Cu-BTC microparticles by the controlled hydrolysis of TBOT. Uniform core-shell Cu-BTC@TiO_2_ microspheres with a mean diameter of 0.6 μm were obtained (Figure 2B,D). The surfaces of the microspheres exhibited a jagged appearance, which may be attributed to small TiO_2_ particles that adhered to the surface and eventually formed an external layer [37]. Thus, titania is not a very dense shell, and some copper element can be detected on the surface, it can be seen Cu mapping images in Figure 3E.

The EDS mapping of Cu-BTC@TiO_2_ (Figure 3) indicated that the yellow (red in TEM) and blue dots with bright color assigned to the Cu and Ti elements, respectively, were homogeneously distributed over the core-shell microparticle. The HRTEM image (Figure 3G) of the region near the interface suggested that the shell was uniformly coated with amorphous TiO_2_. The thickness of the TiO_2_ shell was determined to be about 50 nm. As shown by EDS, the Cu-BTC@TiO_2_ sample exhibited a mixed Cu and Ti signal (Figure 3H). This finding verified that the TiO_2_ was uniformly coated onto the surface of the Cu-BTC cores. Cu and Ti were estimated to be 0.35 atom% and 3.06 atom%, respectively.

**Figure 4:**
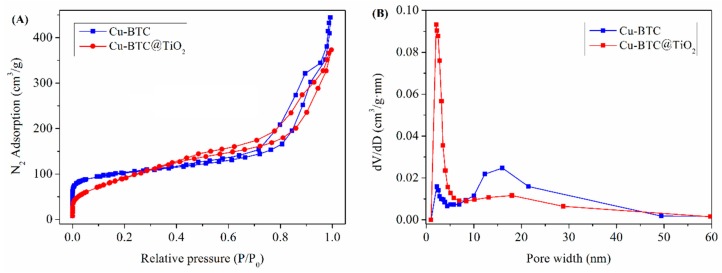
N_2_ adsorption-desorption isotherms (**A**) and pore size distributions on the basis of the Barrett-Joyner-Halenda (BJH) method (**B**) of Cu-BTC and Cu-BTC@TiO_2_.

The surface area and pore distribution of the Cu-BTC and Cu-BTC@TiO_2_ were analyzed using nitrogen adsorption-desorption isotherms (Figure 4). In Figure 4A, the Cu-BTC and Cu-BTC@TiO_2_ samples both presented typical type IV isotherms featuring a large hysteresis loop commonly found in mesoporous materials, suggesting easy transport of the thiophenic S-compounds and products in the pores [38]. The BET specific surface areas of the Cu-BTC and Cu-BTC@TiO_2_ were 733 m^2^/g and 901 m^2^/g, respectively. The Brunauer Emmett Teller (BET) surface area of the Cu-BTC@TiO_2_ was markedly increased, likely because the micropore diameter of the Cu-BTC@TiO_2_ was largely increased by the incorporation of the TiO_2_ into the Cu-BTC, as observed in Figure 4B. The surface area of the Cu-BTC@TiO_2_ was markedly increased, whereas the average pore diameter decreased (17 and 8 nm), indicating the dispersion of TiO_2_ on the surface of the Cu-BTC.

**Figure 5:**
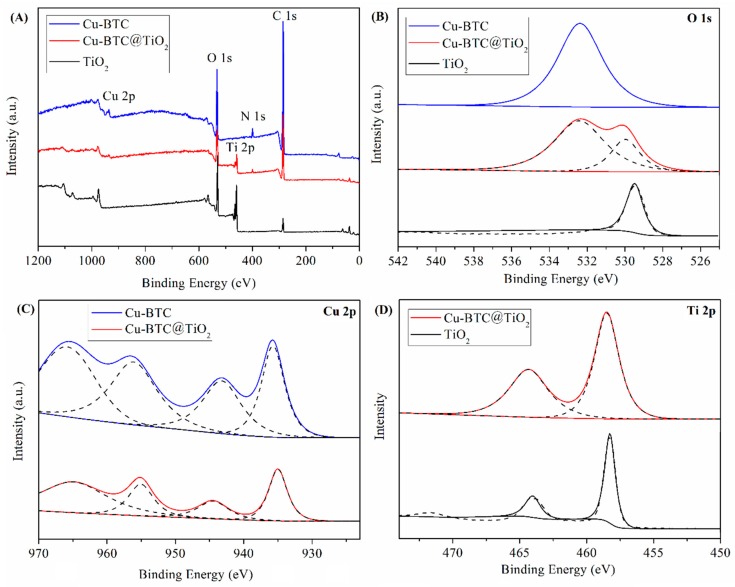
X-ray photoelectron spectroscopy (XPS) survey spectra (**A**) of Cu-BTC, Cu-BTC@TiO_2_ and pureTiO_2_ and narrow scans of the O 1s (**B**), Cu 2p (**C**), and Ti 2p (**D**) regions.

XPS was employed for the surface chemical composition and elemental composition analysis of the Cu-BTC, Cu-BTC@TiO_2_ and pure TiO_2_. The survey spectrum in Figure 5A reveals two sharp peaks at 284.8 and 532.4 eV that may be attributed to the C 1s and O 1s binding energies, respectively. This finding suggested that the two main species in both samples were C and O [39]. Moreover, the peaks at 399.5 and 935.7 eV corresponded to N 1s and Cu 2p of the Cu-BTC and Cu-BTC@TiO_2_. In addition, the XPS spectrum of the Cu-BTC@TiO_2_ and pure TiO_2_ showed new peaks that we may assign to Ti 2p. As shown in the high-resolution spectrum of O (Figure 5B), the binding energy for O 1s appears at approximately 532.4 eV, which is attributed to the presence of O^2−^ ions in the crystalline network of the Cu-BTC [40]. In the case of Cu-BTC@TiO_2_, the asymmetrical O 1s region could be deconvoluted into two peaks at 532.4 and 530.0 eV. The first peak was also associated with O^2−^ ions in the Cu-BTC, whereas the second peak was related to Ti-O bonds in TiO_2_ on the basis of other XPS results on TiO_2_ [41,42]. But the binding energies of pure TiO_2_ at 529.5 eV which decreased 0.5 eV in comparison with the binding energies of Cu-BTC@TiO_2_. Figure 5C presents the XPS spectra of the Cu 2p region. Divalent Cu^2+^ showed a characteristic peak at 935.7 eV, which corresponds with the Cu 2p3/2 and another peak at 956.1 eV that relates to the Cu 2p1/2 [43]. “Shake-up satellite bands” appeared in the 930-970 eV range, except for the 2 characteristic peaks that appeared in the Cu spectra. This observation generally indicates the presence of Cu (II) species [44]. However, the Cu-BTC@TiO_2_ suggested a small change in the Cu 2p XPS spectra. The binding energies of Cu 2p3/2 shifted from 935.7 to 935.0 eV, and those for Cu 2p1/2 shifted from 956.1 to 955.1 eV. These shifts indicated changes in the chemical environment of the Cu. However, this result is still higher than the peak positions for reduced copper species (Cu^0^ peak at 932.5 eV and Cu^+1^ peak at 933.3 eV) [45,46]. It is suggested that Cu maintains in oxidation state which be further confirmed by two satellite peaks at 944.5 eV and 964.9 eV [47]. The FWHM (full width at half maximum) reduced from 3.99 to 3.15 eV. The intensity of the “shake-up satellite” bands was simultaneously reduced. Electron density around atoms exerts a shielding effect on binding energy [48]. Thus, the decrease in binding energy may have resulted from the enhanced electron density around the Cu atoms, suggesting that the Cu^2+^ center accepted lone pair electrons donated by groups of titanium and oxide species, given that the electronic interaction among Cu, Ti and O was attributed to TiO_2_ loading. Figure 5D shows a high-resolution XPS spectra of the Ti 2p. The binding energies of Ti 2p1/2 and Ti 2p3/2 at 464.3 and 458.5 eV, respectively, were contributed by the O-Ti-O bonding with the TiO_2_ [49]. For pure TiO_2_, the binding energies were decreased to 464.0 and 458.3 eV, respectively. These suggested that the TiO_2_-loaded can promote the reduction of Cu^2+^ and the oxidation of Ti^2+^. This implied that Ti oxide species might donate partial electrons to Cu species, the electron transfer between Ti/O and Cu due to the TiO_2_-loaded. Therefore, the electron density and charge distribution in the atoms on the Cu-BTC@TiO_2_ surface may change with the addition of TiO_2_, potentially enhancing the desulfurization activity of the Cu-BTC@TiO_2_. The surface atomic contents of Cu-BTC@TiO_2_ were 0.45% for Cu and 3.68% for Ti. The aforementioned chemical composition agrees with the results of the EDS analysis (Figure 3).

### 3.2. General Analysis of Controlling Factors

**Figure 6:**
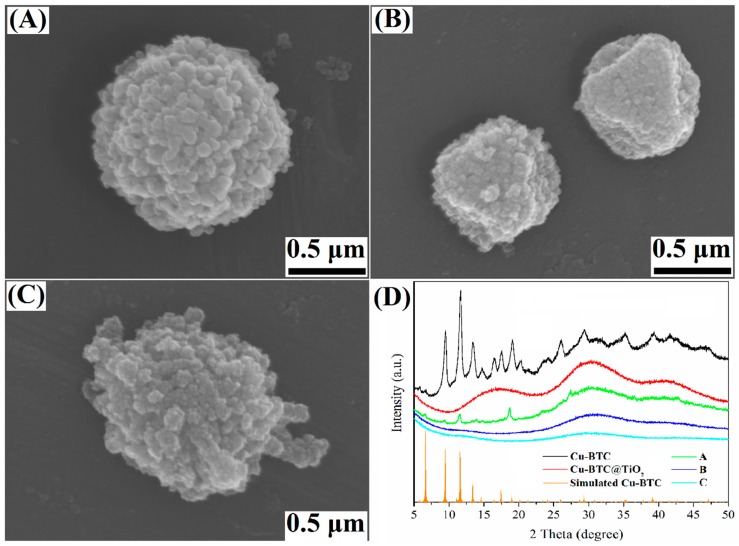
FESEM images of Cu-BTC@TiO_2_ prepared with TiF_4_ (**A**), 0.1 mL of tetrabutylortotitanate (TBOT) (**B**), methanol (**C**), and corresponding XRD patterns (**D**).

Figure 6A–C presents the FESEM images of Cu-BTC@TiO_2_ prepared under different conditions. As shown in Figure 6A, the coverage of the TiO_2_ particulate increases when TiF_4_ is used as a titanium source. When the volume of TBOT decreased to 0.1 mL, the surface of the Cu-BTC microparticles was covered with a thin layer of TiO_2_ particulate (Figure 6B), and the Cu-BTC exhibited a distinct shape. In Figure 6C, the TiO_2_ particulate attached to the Cu-BTC surface appears to be loosely adhering, and the microspheres are not compact. These results suggested that the morphology of the Cu-BTC@TiO_2_ was significantly influenced by the synthesis conditions.

The corresponding XRD patterns of the samples are shown in Figure 6D. The XRD reference pattern simulated from the crystallographic data of Cu-BTC had five typical diffraction peaks assigned to 6.70°, 9.48°, 11.62°, 13.42°, and 17.48° [50], which correspondes with the (200), (220), (222), (400), and (511) planes of the Cu_3_(BTC)_2_ [51]. The Cu-BTC exhibited characteristic diffraction peaks of 9.56°, 11.50°, 13.90°, and 17.60°, and 19.10°, and the main four signals of the samples matched well with the results of simulated Cu-BTC, suggesting that the Cu-BTC was successfully synthesized. XRD patterns. As for Cu-BTC@TiO_2_, the diffraction peak at 5°–50° assigned to the Cu-BTC phase appeared weak and broad. This finding indicates the reduction in crystallinity and TiO_2_ on the surface of Cu-BTC are amorphization, which agrees with the HRTEM results presented in Figure 3G. The Cu-BTC peaks might be covered by the peaks of amorphous TiO_2_ and would be difficult to observe [52]. Thus, the XRD patterns of the Cu-BTC@TiO_2_ showed no crystalline phases, except for the broad peak arising from an amorphous TiO_2_ shell [53]. In all cases, diffractions that could be ascribed to titanium oxides were difficult to observe, indicating that the Ti species was amorphous structure.

### 3.3. Formation Mechanism

**Figure 7:**
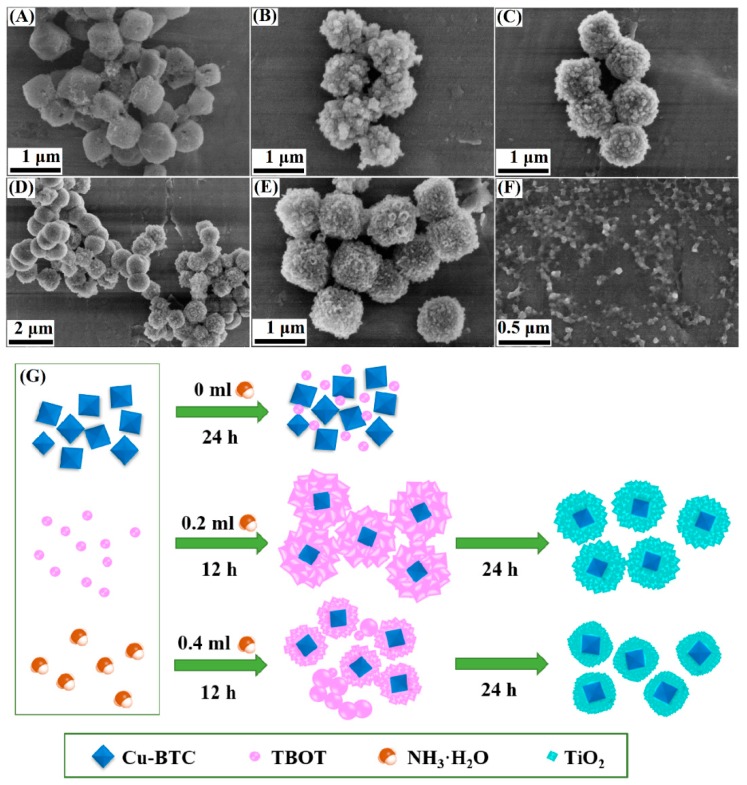
FESEM images of the synthesized Cu-BTC@TiO_2_ with ammonia contents and reaction times of (**A**) 0 mL, (**B**) 2 mL for 12 h, (**C**) 2 mL for 24 h, (**D**) 4 mL for 12 h, (**E**) 4 mL for 24 h, as well as (**F**) isolated TiO_2_ nanoparticles obtained with 0.4 mL of concentrated ammonia for 24 h after the core-shell Cu-BTC@TiO_2_ was removed from the resulting mixtures by centrifugation; (**G**) Schematic of the mechanism underlying the formation of Cu-BTC@TiO_2_ structures.

The coating of the TiO_2_ layer used ammonia as a catalyst for the hydrolysis and condensation of the TBOT. Figure 7A shows that there is very little hydrolysis and condensation of the TBOT because of the absence of ammonia in the solution. Figure 7B shows that with an increase in ammonia content to 0.2 mL, the heterogeneous nucleation of TiO_2_ occurs on the surface of the Cu-BTC microparticles, and uniform microspheric TiO_2_ shells are formed. After the 24 h reaction, the TiO_2_ microspheres continued to grow, forming TiO_2_ particulates (Figure 7C). As shown in Figure 7D, an increase in ammonia content to 0.4 mL prevents the polymerization of titanium oligomers on the surface of the Cu-BTC microparticles to a certain degree; however, homogeneous nucleation and growth can occur in the solution with a reaction time of 12 h. This finding suggested that an excessive amount of ammonia prompted the rapid hydrolysis and condensation of the TBOT. This effect induced the simultaneous occurrence of heterogeneous and homogeneous nucleation and growth, allowing the formation of aggregated structures [36]. When the reaction time was extended to 24 h, the TiO_2_ sphere turned into a TiO_2_ particle and grew on the surface of the Cu-BTC microspheres; also, the TiO_2_ shells thickened to 300 nm (Figure 7E). A milky white suspension appeared when the core-shell Cu-BTC@TiO_2_ microspheres were removed from the mixtures. This appearance suggested the formation of TiO_2_ nanoparticles in the solution and the occurrence of homogeneous nucleation (Figure 7F). A schematic of the mechanism underlying the formation of Cu-BTC@TiO_2_ microspheres is presented in Figure 7G. These results suggested that ammonia significantly controlled the reaction kinetics for the preparation of the uniform core-shell Cu-BTC@TiO_2_ microspheres.

### 3.4. Desulfurization Performance

**Figure 8:**
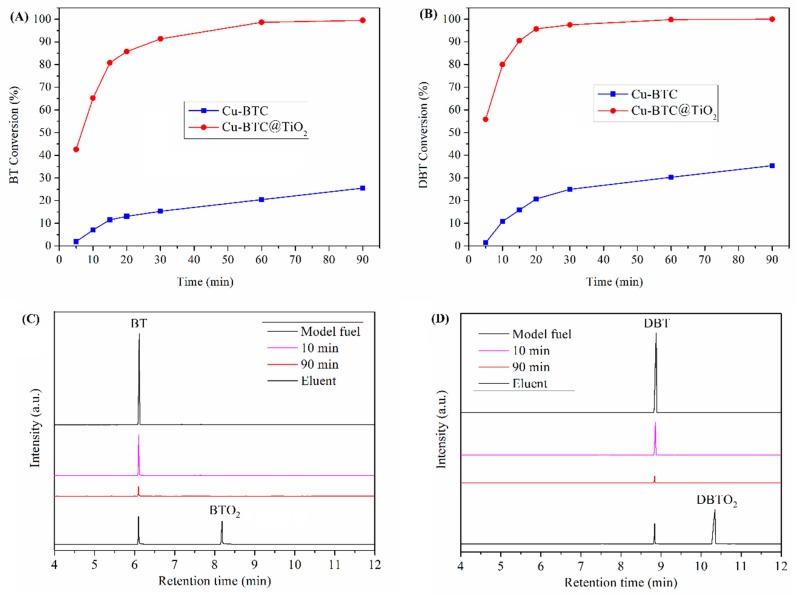
Conversion of BT (**A**) and DBT (**B**) for Cu-BTC and Cu-BTC@TiO_2_. Gas chromatography of the initial model fuel contained BT (**C**) and DBT (**D**), desulfurized fuel samples with adsorption times of 10 and 90 min, and the eluent of the spent Cu-BTC@TiO_2_ (sulfur concentration 1000 ppm, sample amount 100 mg, air flow 60 L/h, reaction temperature 80 °C).

The sulfur removal efficiency of the Cu-BTC@TiO_2_ was evaluated, and the results are shown in Figure 8. With Cu-BTC as the adsorbent for the desulfurization of the model fuel, a BT removal efficiency of less than 30% was obtained. This result indicated that pure Cu-BTC exhibited an extremely low conversion rate, and that the Ti-species in the Cu-BTC played the main role in the catalytic activity. As shown in Figure 8, an increase in reaction time induces considerable increases BT and DBT conversion. Within a 20 min reaction time, BT conversion reached 86%, and DBT conversion reached 96%. These conversions achieved high ADS capacities of 63.76 and 59.39 mg/g. Sulfur contents of BT and DBT in the model oil over Cu-BTC@TiO_2_ were reduced to 140 and 47 ppmw. Cu-BTC@TiO_2_ exhibited BT and DBT conversion that was 6.5 and 4.6 times higher than that of the Cu-BTC, respectively. The Cu-BTC@TiO_2_ obtained similar sulfur removal efficiencies for BT and DBT given a 90 min reaction time (>99%). Ultimately, in the oxidation/adsorption process, less than 10 ppmw BT and less than 20 ppmw DBT was retained in the n-octane phase, which achieved maximal ADS capacities of 73.37 and 62.53 mg/g, respectively. The electron densities of sulfur atoms in the BT and DBT were 5.739 and 5.758, respectively [54], indicating that the catalytic activity of the BT was lower than that of the DBT. 

A report [55,56,57] indicated that a reaction time of at least 30 min would be required to achieve 90% BT and DBT conversion. In the present study, the reaction time required was only 20 min, allowing for the reduction of BT and DBT content in the model oil to 140 and 47 ppmw, with sulfur removal efficiencies of 86% and 95%, respectively. Photocatalysis and adsorption were combined to achieve rapid and deep desulfurization. Therefore, the TiO_2_ shell was the most active site on the surface of the Cu-BTC for BT and DBT oxidation, and the Cu-BTC@TiO_2_ exhibited higher desulfurization efficiency, as compared with Cu-BTC. This finding was attributed to the coexistence and synergistic effects of TiO_2_ and Cu-BTC in the microspheres [58].

Figure 8C,D present the gas chromatography (GC) results of the model fuel containing BT (C) and DBT (D), desulfurization samples at different adsorption times, and the eluent of the used Cu-BTC@TiO_2_ microspheres. As shown in Figure 8C, the peak intensity of BT decreases with adsorption time; however, the chromatograms of desulfurized fuel samples showed no new peaks. To determine how UV light irradiation promoted the sulfur chemistry during desulfurization over Cu-BTC@TiO_2_ microspheres, spent Cu-BTC@TiO_2_ microspheres were washed using a polar solvent (acetonitrile). Figure 8C shows that given a retention time of 8.18 min, a new peak appears, except in the case of the BT. The new peak was benzothiophene sulfone (BTO_2_), as confirmed by GC-MS. Figure 8D shows similar results for DBT. These findings suggest that during desulfurization, UV light irradiation facilitated the oxidation of BT/DBT to BTO_2_/DBTO_2_ under ambient conditions. Consequently, thiophenic compounds were oxidized by the TiO_2_ shell into corresponding sulfones. Meanwhile, intensive absorption occurred between the oxidation products and the inside of the Cu-BTC@TiO_2_ microspheres. These microspheres could simultaneously separate catalysts and products in conditions of deep desulfurization.

### 3.5. The Proposed Desulfurization Mechanism

**Figure 9:**
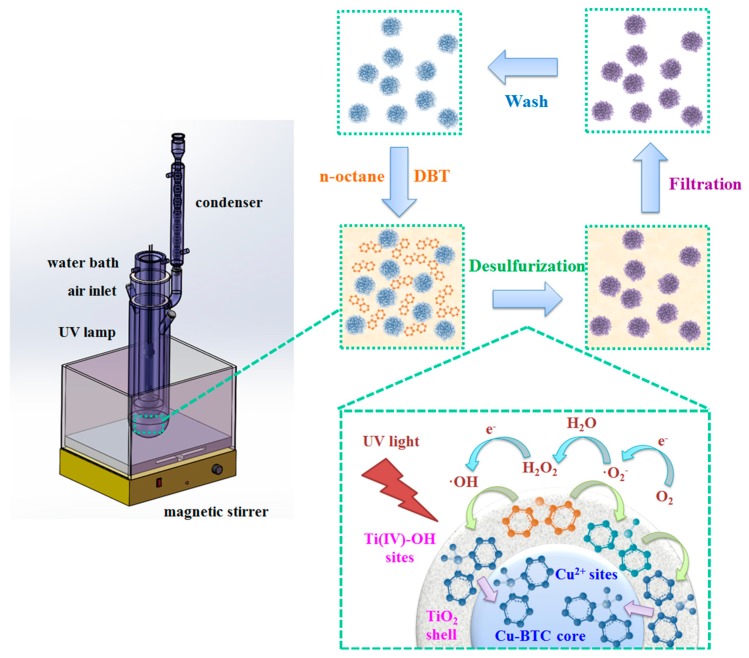
Schematic of the desulfurization mechanism of Cu-BTC@TiO2 microspheres.

This desulfurization system consisted of liquid phases (n-octane and BT/DBT), a solid phase (Cu-BTC@TiO_2_ microspheres), and oxygen. Both BT/DBT oxidation reactions and absorption occurred at liquid-solid interfaces. Figure 9 presents the proposed desulfurization mechanism consisting of seven steps: (1) generation of Bronsted acid Ti(IV)-OH sites under UV irradiation by means of the interaction between chemisorbed H_2_O and bridged-OH groups [26]; (2) enrichment of BT/DBT at the Ti(IV)-OH sites by acid-base interaction; (3) oxidation reaction in the TiO_2_ shell between titanium-based active intermediate species (titanium complexes) and enriched BT/DBT; (4) adsorption of oxidation product sulfones (BTO_2_/DBTO_2_) on the surface of the Cu-BTC core at the metal cation (Cu^2+^) sites by acid-base interaction [59]; (5) extraction of BTO_2_/DBTO_2_ inside the microspheres; (6) simultaneous separation of the Cu-BTC@TiO_2_ microspheres from the solution by simple filtration; and (7) washing of the collected microspheres alternately with acetone and acetonitrile several times to release the sulfones from the microspheres. The washed microspheres may be re-used.

### 3.6. Interaction between Thiophenic Sulfur Compounds and Cu-BTC@TiO_2_ Microspheres

During surface reactions, the TiO_2_ shell exhibited photocatalytic oxidation activity. When O_2_ was used as the oxidant, photo-generated electron transfer to O_2_ on the TiO_2_ was observed. Superoxide anion radicals (O_2_^−^) were formed on this surface when O_2_ reacted with the photo-generated electrons, which could oxidize thiophenic sulfur compounds to sulfoxide or sulfone. The desulfurization was conducted in an open-air system, and limited dissolved oxygen could generate ·OH by reacting ·O_2_^−^ with H^+^. The source of the H^+^ ions could be the hydroxyl groups on the surface and the hydroxyl groups of absorbed water vapor in the air [25]. As the dominant oxidant in photo-oxidation reactions, hydroxyl radicals play a major part in BT/DBT oxidation [42]. Photocatalytic oxidation reactions occurred via the nucleophilic attack of the free electron pairs of sulfur in the BT/DBT by positively charged Ti^4+^ from titanium complexes (Ti-hydroperoxo or Ti-peroxo species [60]) to generate sulfoxide (BTO/DBTO). The resulting sulfoxide was subjected to another oxidation cycle by the feedback of free electrons that attacked another titanium complex in order to produce sulfone (BTO_2_/DBTO_2_). The second oxidation step seemed to progress more quickly than the first one probably because no BTO/DBTO was detected in the final products.

In the absorption step, the Cu-BTC core provided adsorption sites. Cu-BTC could act as a Lewis acid because of its coordinately unsaturated sites (CUSs) that could accept electrons pair from donor molecules. The interaction with Lewis acidic metal ion sites by coordination, and Pearson’s hard and soft acid and bases, explained the adsorption of the basic thiophenic compounds [59]. Thus, soft sulfone bases were highly attracted to soft Lewis acids, such as Cu ions. 

Moreover, the XPS result of the Cu-BTC@TiO_2_ microspheres indicated that the Cu^2+^ center could potentially accept lone pairs of electrons from donating groups of Ti oxide species; it also indicated that the electron density around the Cu atoms was increased, which could be attributed to π-complexation [61]. The interaction of Cu species with the π-electrons of BTO_2_/DBTO_2_ molecules led to the formation of strong π-complex bonds between the Cu and the oxygen atom of the sulfone molecule; this process hastened the adsorption of BTO_2_/DBTO_2_ [62]. Thus, the electronic interaction between Cu and Ti significantly influenced the adsorption capacity of the sulfones.

Therefore, the synergistic effect of Cu-BTC as an adsorbent and TiO_2_ as a catalyst, which captured hydroxyl radicals/activate Th molecules, contributed to the efficient transfer of photo-excited electrons and the oxidation of BT/DBT. In addition, the synergistic effect to which the high desulfurization activity of thiophenic sulfur compounds was attributed increased the rate of the oxidation reaction [24].

**Figure 10:**
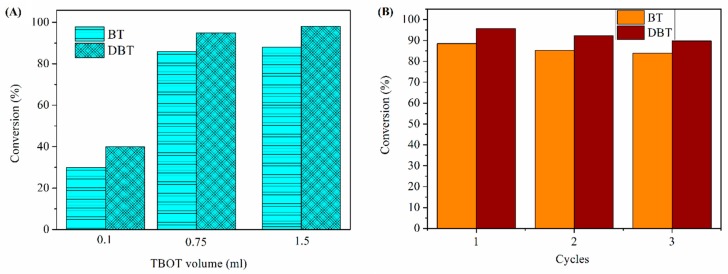
The effect of Ti amount on BT/DBT conversion (**A**) and reusability of Cu-BTC@TiO_2_ (**B**).

The effect of TBOT volume on BT/DBT conversion was showed in Figure 10A. With increase of tetrabutylortotitanate (TBOT) volume from 0.1 mL to 0.75 mL, the BT/DBT conversion increased remarkably. But when TBOT volume increased to 1.5 mL, the BT/DBT conversion almost stable. It is indicated that when adding small amount of Ti, the oxidation activity is too low to transform BT/DBT and Cu-BTC@TiO_2_ microspheres present absorption preformance primarily. When Ti amount exceed 0.75 mL, more Ti cannot coat on the surface of Cu-BTC, thus the BT/DBT conversion was constant. In conclusion, adding 0.75 mL of TBOT volume is the optimum. At the end of the reaction, the Cu-BTC@TiO_2_ microspheres were recovered by simple filtration, washed with acetone, dried, and then subjected to another cycle of desulfurization (Figure 10B). BT removal of Cu-BTC@TiO_2_ decreased to 83.9% after it was used 3 times, and ADS capacity decreased to 31.17 mg/g. DBT removal of Cu-BTC@TiO_2_ was reduced to 89.8% after it was used 3 times, and ADS capacity decreased to 54.55 mg/g. These results indicated that the deactivation of Cu-BTC@TiO_2_ microspheres could be inhibited by the core-shell structure; such inhibition ensures good recyclability. In addition, the catalysts regenerated by acetone could improve in repeated-use performance.

### 3.7. Kinetic Calculations

**Figure 11:**
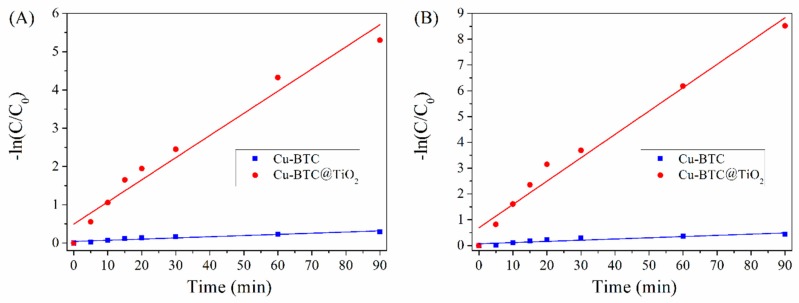
Effect of time on the plots of the pseudo-first-order kinetics of BT (**A**) and DBT (**B**) over Cu-BTC and Cu-BTC@TiO_2_.

The changes in −ln(C/C_0_) versus time (t) for the Cu-BTC and Cu-BTC@TiO_2_ are presented in Figure 11. The linear relationships obtained confirm the pseudo-first-order reaction kinetics proposed. The reaction equation is expressed as follows [41]:

−ln(C/C_0_) = kt (2)

where C_0_ and C are the initial and reaction time concentrations of BT/DBT, respectively; k is the apparent rate constant, and t is the desulfurization time. The slope of the linear fitted plot indicates the apparent rate constant for desulfurization. Table 1 presents the values of k obtained in the present study; the results show that Cu-BTC@TiO_2_ significantly and positively influences the desulfurization of BT/DBT. The k value of Cu-BTC@TiO_2_ was almost 100-fold higher than that of the Cu-BTC under the same reaction conditions. This difference demonstrates the considerably higher desulfurization efficiency of BT/DBT over Cu-BTC@TiO_2_ versus that of Cu-BTC. The linear coefficient (R^2^) was also higher than 0.95 for Cu-BTC@TiO_2_. This difference suggests that the desulfurization of BT/DBT followed pseudo-first-order kinetics, and that the Cu-BTC@TiO_2_ creates efficient desulfurization microspheres for BT/DBT.

Higher rate constants were obtained for DBT than for BT. These results indicate that DBT could be more easily desulfurized than BT. The performance of Cu-BTC and Cu-BTC@TiO_2_ in BT/DBT desulfurization generally improved as electron density on the S-atom increased [63]. The rate constants for BT/DBT over Cu-BTC and Cu-BTC@TiO_2_ exhibited a high correlation with the corresponding electron densities, which were 5.739 and 5.758, respectively, as calculated by Otstuki et al. [54]. 

## 4. Conclusions

Cu-BTC@TiO_2_ microspheres exhibiting photocatalysis-assisted adsorptive desulfurization were successfully synthesized. The microspheres combined TiO_2_ photocatalysts and a Cu-BTC adsorbent that selectively removed thiophenic S-compounds by integrating with selective oxidation and solid adsorption. The diameter of the Cu-BTC@TiO_2_ microspheres was approximately 0.6 μm. FESEM, and HRTEM images revealed the jagged surface of the microspheres, which suggested the formation of an external layer by the adhesion of small TiO_2_ particles. A mixed Cu and Ti signal was obtained from the Cu-BTC@TiO_2_ sample by EDS mapping. BET showed that Cu-BTC and Cu-BTC@TiO2 obtained large increases in surface area and significant reductions in the average pore diameter (16.64 and 8.04 nm, respectively). These results indicated the dispersion of TiO_2_ on the surface of the Cu-BTC. The XPS results showed that adding TiO_2_ induced changes in the electron density and charge distribution of the atoms on the surface of the Cu-BTC@TiO_2_. These changes could potentially enhance the desulfurization activity of the Cu-BTC@TiO_2_. In the coating process of the TiO_2_ layer, carefully controlled ammonia content played a key role in controlling the reaction kinetics to construct uniform core-shell Cu-BTC@TiO_2_ microspheres. The as-synthesized Cu-BTC@TiO_2_ microspheres showed excellent desulfurization performance. Within a 20 min reaction time, BT conversion reached 86%, and DBT conversion was as high as 95%. These results achieved high ADS capacities of 63.76 and 59.39 mg/g, respectively. Sulfur contents in the model oil were reduced to 140 and 47 ppmw. Cu-BTC@TiO_2_ exhibits BT and DBT conversion rates that were 6.5 and 4.6 times higher than that of Cu-BTC, respectively. The proposed 7-step desulfurization mechanism and the synergistic effect of Cu-BTC (as an adsorbent) and TiO_2_ (as a catalyst), which capture hydroxyl radicals/activate molecules, contributed to the efficient transfer of photo-excited electrons and to the oxidation of BT/DBT. In addition, the aforementioned mechanism and synergistic effects, to which the high desulfurization activity of the thiophenic sulfur compounds could be attributed, increased the rate of the oxidation reaction. Kinetic studies demonstrated that the desulfurization kinetics of BT/DBT followed a pseudo-first-order reaction. The used microspheres could be readily regenerated using a solvent washing method. The microspheres could also be reused at least 3 times. The results of this study demonstrate that the combination of photocatalysis and adsorption could achieve rapid and deep desulfurization. The study also shows the potential use of Cu-BTC@TiO_2_ microspheres as an adsorbent for the removal of thiophenic sulfurs from fuels.

## Figures and Tables

**Table 1:** Pseudo-first-order rate constants and correlation factors of the desulfurization of BT/DBT ^1^.

Catalysts	BT		DBT	
	k (min^−1^)	R^2^	k (min^−1^)	R^2^
Cu-BTC	0.0424	0.9230	0.0680	0.9558
Cu-BTC@TiO_2_	0.4953	0.9598	0.6867	0.9735

^1^ k, first-order rate constant; R^2^, correlation parameter.
